# Supplementary material for: The Small RNA Universe of Capitella teleta
Source: Front Mol Biosci. 2022 Feb 25;9:802814. doi: 10.3389/fmolb.2022.802814 (PMC8915122; doi:10.3389/fmolb.2022.802814)
Supplement: Supplementary file 1 [file DataSheet1.ZIP › Supplement/known/CAPTEscaffold_222_14878.pdf]

Provisional ID	:	CAPTEscaffold_222_14878
Score total	:	40279.2
Score for star read(s)	:	3.9
Score for read counts	:	40269.2
Score for mfe	:	1.6
Score for randfold	:	1.6
Score for cons. seed	:	3
Total read count	:	78998
Mature read count	:	78844
Loop read count	:	1
Star read count	:	153

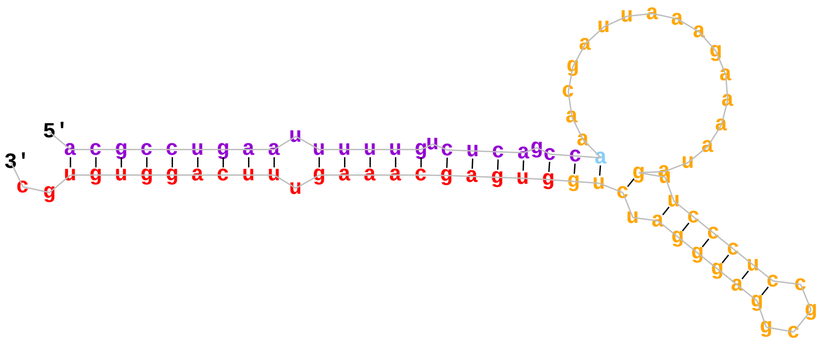

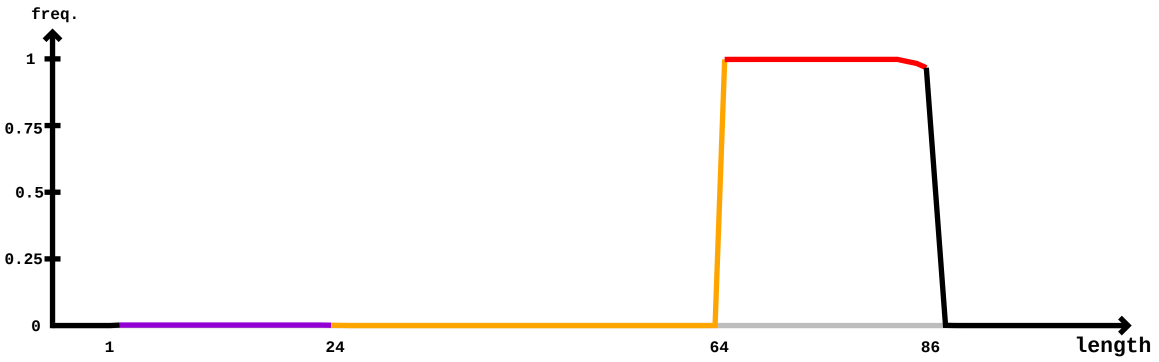

Star **Mature**

```
5'- caacgacgccugaauuuuugucucagccaaacgauuaaagaaaauaguucccuccgcggagggaucuggugagcaaaguuucaggugugcuugcggacugcacugccuuc  -3'  obs
    caacgacgccugaauuuuugucucagccaaacgauuaaagaaaauaguucccuccgcggagggaucuggugagcaaaguuucaggugugcuugcggacugcacugccuuc       exp
    caacgacgccugaauuuuugucucagccaaacgauuaaagaaaauaguucccuccgcggagggaucuggugagcaaaguuucaggugugcuugcggacugcacugccuuc       known
    .....((((((((.(((((.((((.(((...................(.((((((....)))))).))))))))))))).))))))))....((((......))))....     reads   mm   sample
    .....acgccugaauuuuugucuca.....................................................................................     1       0    seq
    .....acgccugaauuuuugucucag....................................................................................     5       0    seq
    .....acgccugaauuuuugucucagc...................................................................................     44      0    seq
    .....acgccugaauuuuugucucagcc..................................................................................     57      0    seq
    .....acgccugaauuuuugucucagcca.................................................................................     37      0    seq
    .....acgccugaauuuuugucucagccU.................................................................................     7       1    seq
    .....Gcgccugaauuuuugucucagcca.................................................................................     1       1    seq
    .....acgccugaauuuuugucucagccaa................................................................................     1       0    seq
    .............................aacgauuaaagaaaauaguucccuccgcg....................................................     1       0    seq
    ..................................................................uCgugagcaaaguuucaggugugc....................     1       1    seq
    ...................................................................Agugagcaaaguuucaggugugc....................     1       1    seq
    ...................................................................Cgugagcaaaguuucaggugugc....................     6       1    seq
    ....................................................................gugagcaaaguuucaggu........................     4       0    seq
    ....................................................................gugaCcaaaguuucaggug.......................     1       1    seq
    ....................................................................gugagcaaaguuucagCug.......................     1       1    seq
    ....................................................................gugagcaaaguuucaAgug.......................     1       1    seq
    ....................................................................gugagcaaaguuuUaggug.......................     1       1    seq
    ....................................................................guCagcaaaguuucaggug.......................     1       1    seq
    ....................................................................gugagcaaaguuucaggug.......................     598     0    seq
    ....................................................................gugagcaaaguuucaggGg.......................     2       1    seq
    ....................................................................gAgagcaaaguuucaggug.......................     2       1    seq
    ....................................................................gugagcaaaguuuUaggugu......................     2       1    seq
    ....................................................................gNgagcaaaguuucaggugu......................     1       1    seq
    ....................................................................gugagcaaaguuucaggugG......................     14      1    seq
    ....................................................................gugagcaaaguuucaggugu......................     566     0    seq
    ....................................................................gugagcaaagAuucaggugu......................     1       1    seq
    ....................................................................gGgagcaaaguuucaggugu......................     1       1    seq
    ....................................................................gugagcaaaguuucaggugC......................     2       1    seq
    ....................................................................gugagcaaaguuucaAgugu......................     1       1    seq
    ....................................................................gAgagcaaaguuucaggugu......................     1       1    seq
    ....................................................................gugagcaaaguuucaggugGg.....................     1       1    seq
    ....................................................................Uugagcaaaguuucaggugug.....................     3       1    seq
```

Star **Mature**

caacgacgccugaauuuuugucucagccaaacgauuaaagaaaauaguucccuccgcggagggaucuggugagcaaaguuucaggugugcuugcggacugcacugccuuc

....gugagcaaaguuGcaggugug.....................	1	1	seq
....gugagcaaaguuucGggugug.....................	1	1	seq
....guAagcaaaguuucaggugug.....................	3	1	seq
....gugagcaaaguuucaggugug.....................	1246	0	seq
....gugagcaaagAuucaggugug.....................	1	1	seq
....gugagcaaaguuuUaggugug.....................	2	1	seq
....gugagcaaaguuucagguguU.....................	3	1	seq
....gugagcaaaguuucagguguC.....................	1	1	seq
....gugagcaaaguuucagguUug.....................	1	1	seq
....gugagcaaaguuucaggGgug.....................	1	1	seq
....gugagcaaaguCucaggugug.....................	1	1	seq
....gugagcaUaguuucaggugug.....................	1	1	seq
....gAgagcaaaguuucaggugug.....................	3	1	seq
....gugagcaaaguuucagAugug.....................	1	1	seq
....gugagcGaaguuucaggugugc....................	9	1	seq
....gugagcaaaguuucagguguCc....................	2	1	seq
....gugagcaaaguuuAaggugugc....................	14	1	seq
....gugagcaGaguuucaggugugc....................	6	1	seq
....gugagcaaaguAucaggugugc....................	21	1	seq
....gugagcaaaguuucagAugugc....................	11	1	seq
....gugaCcaaaguuucaggugugc....................	2	1	seq
....gugagcaaUguuucaggugugc....................	1	1	seq
....gugagcaaaguuucaUgugugc....................	7	1	seq
....gugagcaaaguuGcaggugugc....................	2	1	seq
....gugagcaaaguuucaggugugc....................	73409	0	seq
....gugagcaaaguuucaggugugG....................	208	1	seq
....gugagcaUaguuucaggugugc....................	5	1	seq
....gugGgcaaaguuucaggugugc....................	10	1	seq
....gugagcaCaguuucaggugugc....................	1	1	seq
....gugagcaaagGuucaggugugc....................	8	1	seq
....gCgagcaaaguuucaggugugc....................	7	1	seq
....gugagcaaaguuucaAgugugc....................	35	1	seq
....gugagcaaaAuuucaggugugc....................	1	1	seq
....gugagcaaaUuuucaggugugc....................	3	1	seq
....gugaUcaaaguuucaggugugc....................	6	1	seq
....gugagcaaaguuucagguguAc....................	19	1	seq
....gugagNaaaguuucaggugugc....................	2	1	seq
....gugagGaaaguuucaggugugc....................	1	1	seq
....guAagcaaaguuucaggugugc....................	77	1	seq
....gugagAaaaguuucaggugugc....................	30	1	seq
....gugagcaaGguuucaggugugc....................	11	1	seq
....gugagcaaaguuucGggugugc....................	29	1	seq
....gugagcaaaguuucagguCugc....................	5	1	seq
....gugagcaaaguCucaggugugc....................	10	1	seq
....gugagcaaaguuCcaggugugc....................	12	1	seq
....gugagcUaaguuucaggugugc....................	4	1	seq
....gugagcaaaguuucaggugCgc....................	7	1	seq
....gugagcaaaguuucaggCgugc....................	3	1	seq
....gugagcaaaguuucUggugugc....................	12	1	seq
....gugagcaaaguuucaggugugN....................	1	1	seq
....guUagcaaaguuucaggugugc....................	8	1	seq
....gugCgcaaaguuucaggugugc....................	1	1	seq
....Nugagcaaaguuucaggugugc....................	13	1	seq
....gugagcaaaguuucaggugAgc....................	28	1	seq
....Uugagcaaaguuucaggugugc....................	132	1	seq
....gugagcaaaguuucaCgugugc....................	1	1	seq
....gugagcaaaCuuucaggugugc....................	2	1	seq
....gAgagcaaaguuucaggugugc....................	50	1	seq
....guCagcaaaguuucaggugugc....................	9	1	seq
....gugagcaaaguuucagguAugc....................	5	1	seq
....gugagcaaaguuAcaggugugc....................	17	1	seq
....gugagcaaaguuucagCugugc....................	6	1	seq
....gugagcaaaguuucaggugugA....................	51	1	seq
....gugagcaaaguuucagguUugc....................	1	1	seq
....Cugagcaaaguuucaggugugc....................	14	1	seq
....gugagcaaaguuucagUugugc....................	3	1	seq
....gugagcaaaguuucaggugGgc....................	1	1	seq
....gugagcaaaNuuucaggugugc....................	1	1	seq
....gugagcaaaguuucaggugugU....................	436	1	seq
....gugagcaaagAuucaggugugc....................	15	1	seq

```
     Star                                                              Mature
caacgacgccugaauuuuugucucagccaaacgauuaaagaaaauaguucccuccgcggagggaucuggugagcaaaguuucaggugugcuugcggacugcacugccuuc
....................................................................gNgagcaaaguuucaggugugc....................    4     1    seq
....................................................................gugagcaaaguuucCggugugc....................    1     1    seq
....................................................................gugagcaaaguGucaggugugc....................    1     1    seq
....................................................................gugagcaaaguuuGaggugugc....................    11    1    seq
....................................................................gugagcaaagCuucaggugugc....................    5     1    seq
....................................................................gGgagcaaaguuucaggugugc....................    16    1    seq
....................................................................gugUgcaaaguuucaggugugc....................    9     1    seq
....................................................................gugagUaaaguuucaggugugc....................    7     1    seq
....................................................................gugagcaaaguuucaggAgugc....................    22    1    seq
....................................................................gugaAcaaaguuucaggugugc....................    10    1    seq
....................................................................Augagcaaaguuucaggugugc....................    17    1    seq
....................................................................gugagcaaaguuuUaggugugc....................    99    1    seq
....................................................................gugagcaaaguuucagguguUc....................    7     1    seq
....................................................................gugagcaaaguuucaggugugcG...................    4     1    seq
....................................................................gugagcaaaguuucaggugugUu...................    8     1    seq
....................................................................gGgagcaaaguuucaggugugcu...................    1     1    seq
....................................................................gugagcaaaguuucaggugugcA...................    97    1    seq
....................................................................gugagcaaaguuucaUgugugcu...................    1     1    seq
....................................................................gugagcaaaguuucGggugugcu...................    1     1    seq
....................................................................gugagcaaaguuucaggugugcC...................    23    1    seq
....................................................................gugagcaaaguuucaggugugcu...................    1156  0    seq
....................................................................gugagcaaaguuucagguguAcu...................    1     1    seq
....................................................................gugagcaaaguuucaAgugugcu...................    2     1    seq
....................................................................gugagcaaaguuucaggugugAu...................    3     1    seq
....................................................................gugagcaaaguuucaggugugcuA..................    3     1    seq
....................................................................gugagcaaaguuucaggugugcCu..................    23    1    seq
....................................................................gugagcaaaguuucaggugugcAu..................    11    1    seq
....................................................................gugagcaaaguuucaggugugUuu..................    2     1    seq
....................................................................gugagcaaaguuucaggugugcuu..................    20    0    seq
....................................................................gugagcaaaguuucaggugugcuuU.................    6     1    seq
.....................................................................ugagcaaaguuucaggugugc....................    4     0    seq
......................................................................gagcaaaguuucaggugugc....................    3     0    seq
.......................................................................agcaaaguuucaggugugc....................    1     0    seq
........................................................................gcaaaguuucaggugugc....................    2     0    seq
```
